# Cell to Cell Variability of Radiation-Induced Foci: Relation between Observed Damage and Energy Deposition

**DOI:** 10.1371/journal.pone.0145786

**Published:** 2016-01-04

**Authors:** Gaëtan Gruel, Carmen Villagrasa, Pascale Voisin, Isabelle Clairand, Marc Benderitter, Jean-François Bottollier-Depois, Joan Francesc Barquinero

**Affiliations:** Department of Human Health Radiation Protection, Institut de Radioprotection et de Sûreté Nucléaire (IRSN), Fontenay aux Roses, France; ENEA, ITALY

## Abstract

Most studies that aim to understand the interactions between different types of photon radiation and cellular DNA assume homogeneous cell irradiation, with all cells receiving the same amount of energy. The level of DNA damage is therefore generally determined by averaging it over the entire population of exposed cells. However, evaluating the molecular consequences of a stochastic phenomenon such as energy deposition of ionizing radiation by measuring only an average effect may not be sufficient for understanding some aspects of the cellular response to this radiation. The variance among the cells associated with this average effect may also be important for the behaviour of irradiated tissue. In this study, we accurately estimated the distribution of the number of radiation-induced γH2AX foci (RIF) per cell nucleus in a large population of endothelial cells exposed to 3 macroscopic doses of gamma rays from ^60^Co. The number of RIF varied significantly and reproducibly from cell to cell, with its relative standard deviation ranging from 36% to 18% depending on the macroscopic dose delivered. Interestingly, this relative cell-to-cell variability increased as the dose decreased, contrary to the mean RIF count per cell. This result shows that the dose effect, in terms of the number of DNA lesions indicated by RIF is not as simple as a purely proportional relation in which relative SD is constant with dose. To analyse the origins of this observed variability, we calculated the spread of the specific energy distribution for the different target volumes and subvolumes in which RIF can be generated. Variances, standard deviations and relative standard deviations all changed similarly from dose to dose for biological and calculated microdosimetric values. This similarity is an important argument that supports the hypothesis of the conservation of the association between the number of RIF per nucleus and the specific energy per DNA molecule. This comparison allowed us to calculate a volume of 1.6 μm^3^ for which the spread of the specific energy distribution could explain the entire variability of RIF counts per cell in an exposed cell population. The definition of this volume may allow to use a microdosimetric quantity to predict heterogeneity in DNA damage. Moreover, this value is consistent with the order of magnitude of the volume occupied by the hydrated sugar-phosphate backbone of the DNA molecule, which is the part of the DNA molecule responsible for strand breaks.

## Introduction

DNA double-strand breaks (DSB) are critical lesions that produces a variety of radiobiological effects [1,2]. Understanding the interactions of different radiation qualities with DNA requires an accurate quantification of the yields of DNA DSBs. Among the techniques used to measure DSB yields in mammalian cells are: neutral sedimentation gradients, filter elution, pulsed field gel electrophoresis techniques (PFGE) [3], and more recently, immunofluorescence analysis of nuclear foci of DSB-signalling proteins, including phosphorylation of H2AX at Ser139 (alias gamma H2AX) [4], phosphorylation of ATM [5], or localization of MRE11 [6] or 53BP1 [7]. Specific antibodies enable the visualization of discrete nuclear foci (RIF for radiation induced foci) at DSB sites. Initial studies have shown a close correlation between the number of RIF and the number of DSBs expected after gamma-ray exposure and several have observed a linear relation between the mean per-cell RIF count and the mean macroscopic absorbed dose of photon radiation[8–10]. In general, these studies have assumed homogeneous cell irradiation, that is, that all cells receive the same amount of energy. The level of DNA lesions is therefore generally determined by averaging it over the entire population of exposed cells. Intercell variation in the number of RIF is used to measure a kind of uncertainty associated with biological and methodological phenomena, and the average level of damage is the value usually considered to be responsible for the final biological effect. Nevertheless, at the micrometric and nanometric (molecular) scales, the distribution of the energy deposited by ionizing radiation has a spread that is due to fluctuations in both the number of tracks passing the target and the energy deposition per track [11–14]. These fluctuations depend on the radiation quality and the target volume. Thus, the assessment of a stochastic phenomenon such as ionizing radiation by the measurement of an average effect cannot provide an adequate understanding of some aspects of the cellular response to this radiation. The RIF count variation among the cells implies that different quantities of damage have been signalled for these different cells, and hence that there exist cells for which the biological effect may be different from those with the mean level of damage. This point is important, both in the context of cancer treatment where the survival of just a few cells may induce relapse but also in analysing the processes underlying tissue response to low-dose exposures or low fluence. Accordingly, the evaluation of this RIF variance between cells appears to be as important as the mean number of RIF for assessing the biological significance of radiation exposure.

In this work, we accurately measured the variability of RIF counts in each nucleus in a population of cells exposed to three different macroscopic doses, in order to evaluate the portion of this variability associated with the microdosimetric variance calculated for the different target volumes and subvolumes in which RIF can be generated.

## Materials and Methods

### Cell Culture and Irradiation

The human umbilical vein endothelial cells (HUVEC) we used were purchased from the Lonza Group (ref. C2519A, lot. 87758) and isolated by Lonza from human tissue (from 3 females and 1 male) donated after permission was obtained for its use in research applications by informed consent or legal authorization. Lonza holds donor consent and legal authorization that provides permission for all research use. Hence, institutional review board or ethics committee approval was not necessary. The supplier states that it followed established ethical practices of United States donation and transplantation organizations. In addition, all experiments complied with French law (Act no. 2004–800) on bioethics. All cells tested negative for mycoplasma, bacteria, yeast and fungi. Cell lots and donors were tested and negative for HIV-1, hepatitis B and hepatitis C. The HUVEC were cultured at 37°C, with 95% humidity and 5% CO_2_ in EGM-2 media optimized for the proliferation of endothelial cells and supplemented with 5% fetal bovine serum, hydrocortisone, hFGF-B, VEGF, R3-IGF-1, ascorbic acid, hEGF, gentamicin and amphotericin-B (EGM-2MV BulletKit, Lonza). For experiments, cells were seeded on glass in 1-well Nunc^®^ Lab-Tek^®^ II chamber slide systems (Thermo Fisher Scientific). The cells were grown until they formed a monolayer (approximately 75 to 85% confluent) and then exposed to gamma radiation from a 145817 GBq source (nominal activity 22/02/2002) of ^60^Co radiation (ICO 4000 IRSN facility at Fontenay-aux-Roses, France), at a dose rate of 1.3 Gy.min^-1^. The uncertainty for the delivered dose was estimated to be 6%. Two different platforms were used for the X-ray exposures to corroborate the results obtained with ^60^Co: an Eleckta Synergy Platform (linac accelerator) was used to deliver radiation with a maximal energy of 4 MeV (X-rays 4 MVp) at a dose rate of 1.1 Gy.min^-1^ and with an uncertainty for the delivered dose estimated at 7%, and the X-RAD 320 X-ray system (XPI) delivered radiation with a maximal energy of 200 kVp, at a dose rate of 0.54 Gy.min^-1^, and with an uncertainty for the delivered dose below 2%.

### Immunostaining

HUVEC were immunostained after exposure to sham, 0.5 Gy, 1 Gy and 2 Gy of ^60^Co irradiation. Briefly, 30 min after irradiation, cells were fixed with 4% paraformaldehyde in PBS for 15 min, washed with PBS (Life Technologies), permeabilized in 0.5% Triton X-100 solution (Sigma Aldrich) for 5 min, and then washed 3 more times with PBS. The cells were then incubated for 1 h with primary antibodies, either a mix of mouse IgG1 monoclonal anti-phospho-histone H2AX (Ser139) antibody (clone JBW301, Upstate) and/or rabbit polyclonal anti-KI67 (Life Technologies). Cells were then washed 3 more times and incubated for 1 h with a secondary antibody, i.e., a mix of goat anti-mouse IgG1 (γ1) coupled to Alexa Fluor® 488 (2 mg.mL^-1^, Life Technologies) and/or goat anti-rabbit IgG (H+L) coupled to Texas Red®-X (2 mg.mL^-1^, Life Technologies). Cells were washed as described above before applying ProLong® Gold Antifade mountant with DAPI (Life Technologies).

### Image Acquisition and Analysis

Cell images were visualized with a ScanR platform (Olympus), which comprises an inverted microscope IX81 (Olympus) equipped with a motorized SCAN IM IX2 stage (Märzhäuser) and an MT20 fluorescence illumination system with a fast filter wheel. Images were acquired with a UPLSAPO 100XO oil immersion objective (Olympus) with an NA of 1.4 and an ORCA-R2 CCD camera (Hamamatsu). The image pixel size was 0.064 μm. The limit of resolution based on the NA of the objective in the Alexa Fluor® 488 channel was 0.2 μm (Rayleigh limit). Images were captured so that intensities for a given experiment were within the 12-bit linear range. Image analysis was performed with Scan^R analysis software. The edge-based segmentation algorithm implemented in the software was used to detect main objects (*i*.*e*., nuclei) and subobjects (*i*.*e*., foci). Different kinds of parameters were measured on each detected object and subobject. Those used for the data analysis were mainly the area, circularity, and integrated intensity of DAPI, Alexa Fluor® 488 and Texas Red®-X. Non-cycling cells were selected with a “flow cytometry like” analysis. A first selection based on areas and circularities of the nuclei was done through the definition of an adequate region on the corresponding scatter plot. This step allowed us to consider only isolated nuclei and to remove from the analysis objects corresponding to clusters of nuclei. A second region, based on the integrated intensity levels of DAPI and Alexa Fluor® 488, measured for each nucleus, was then done to isolate nuclei in the G0/G1 phase of the cell cycle. RIF in the objects within the gate formed by the intersection of the two regions were then analysed.

### Specific Energy Distribution Calculation

Specific energy was calculated only for the ^60^Co gamma radiation exposure. The energy absorbed by a specific target follows a frequency distribution ƒ(z) where z is a stochastic quantity corresponding to the energy ε imparted per mass *m* in the target of volume V. The macroscopic dose *D* corresponds to the expected value of the distribution ƒ(z) [11–14].

The probability of obtaining a specific energy z in the target is the result of two stochastic phenomena: the probability of ν tracks passing the target and the probability that these ν tracks deposit a specific energy equal to z.

$$f(z,D)=\sum_{\nu =0}^{\infty }p(\nu )\cdot f_{\nu }(z)$$ (1)

where, for small doses, the number of tracks is distributed following a Poisson distribution of mean value n,

$$p\left(\nu \right)=e^{-n}\frac{n^{\nu }}{\nu !}.$$

For n>20, the Poisson distribution can be approximated well by a normal distribution of mean value n and standard deviation √n [15]. This is true in this work because for ^60^Co irradiation this condition is met in typical cell nucleus volumes for D> 0.2 Gy.

The function ƒ_v_(z) can be calculated as the convolution of the single-track frequency distribution ƒ_1_(z):

$$f_{\nu }\left(z\right)\;=\;\int_{0}^{z_{max}}f_{\nu -1}(z-z^{\prime })\cdot f_{1}(z^{\prime })\;dz\prime \;.$$ (2)

This convolution rapidly converges to a normal distribution of the form:

$$N\left(\nu \cdot \bar{z_{1}},\sqrt{\nu }\cdot {SD}_{z_{1}}\right),$$

wherez_1_ and SDz_1_ are respectively the mean value and the standard deviation of the single track frequency distribution. Thus, the convolution of both distributions in Eq 1 can be approximated to a normal distribution:

$$N(n\cdot \bar{z_{1}};\sqrt{n\cdot \left({{\bar{z}}_{1}}^{2}+{{SD}_{z_{1}}}^{2}\right)})$$

Accordingly, and knowing thatz_1_ and SDz_1_ are both functions of the volume, we can calculate the parameters *k*_*1*_ and *k*_*2*_ needed to express the relative standard deviation of the specific energy distribution of the ^60^Co irradiation as a function of the macroscopic dose D and the volume of the target V [15]:

$${SD}_{z}^{rel}=\frac{k_{1}}{V^{k_{2}}\sqrt{D}}$$ (3)

In this work, we used this relation and the k_1_ and k_2_ values obtained by Villegas *et al*. [15] to calculate the specific energy distributions expected in different volumes related to the endothelial cell population irradiated with the ^60^Co source at the IRSN facility.

## Results

### Characterization of HUVEC Cells for RIF Analysis

The mean number of DSBs per cell induced by a given dose of gamma rays is proportional to the DNA content of the cells. Accordingly, it was necessary to use a cell population with a homogeneous DNA content, to be able to interpret the intercellular variability of RIF count per cell and be certain that intercellular variations in the initial DNA content were not the source of this post-exposure RIF variability. Preliminary analyses were conducted to characterize the HUVEC cells and ensure the robustness of this aspect of our experimental approach.

Because *in vitro* cell culture may induce genomic instability, HUVEC primary cells were used at low passage, and their cytogenetic state was evaluated by M-FISH during passages 2 (P2) and 4 (P4). Of the 78 cells analysed in P2, 51 were female (65%) and 27 male (35%), and only two were abnormal, both with trisomy ((47, XY, +12) and (47, XX, +18)). In P4, 105 cells were analysed, 80 female (76%) and 25 male (24%). Again, two abnormal cells were observed, one trisomy (47, XX, +11) and one cell with two non-reciprocal translocations and one double insertion (46, XY, t(14;5), t(14;Y), ins (2;22;3)). No clonal abnormality was observed, and the proportions of males and females obtained by cytogenetic analysis were consistent with the initial proportions of the pool of cells (3 females and 1 male).

**Fig 1:**
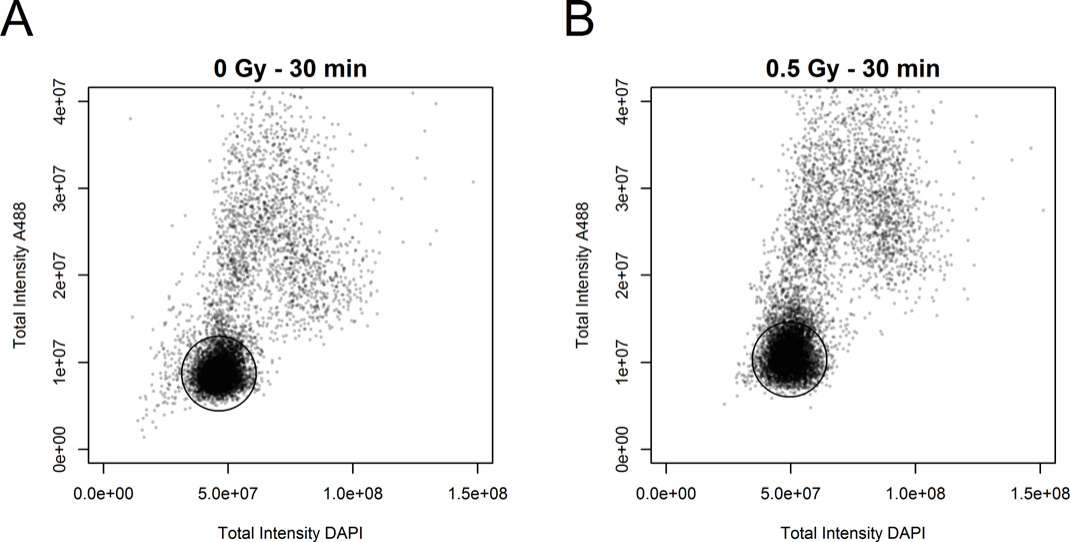
Scatter plots of the integrated intensities of DAPI and Alexa Fluor® 488. The integrated intensities of DAPI and Alexa Fluor® 488 correspond respectively to the DNA content and level of gamma-H2AX in the whole nucleus of each analysed cell. Black circles indicate a well-defined population of nuclei mainly in the G0/G1 phase of the cell cycle. (A) Non-irradiated condition. (B) Irradiated condition, 0.5 Gy.

**Fig 2:**
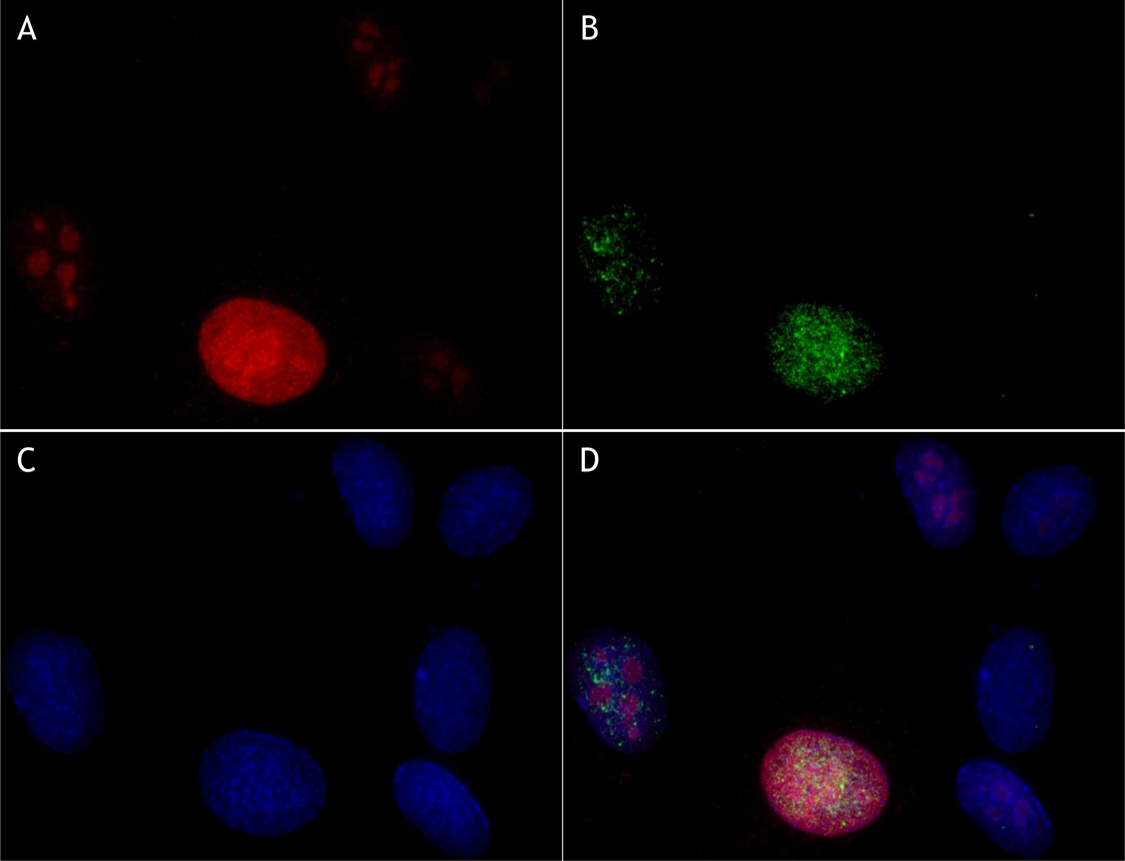
Co-labelling by immunofluorescence of gamma-H2AX and KI67 in non-irradiated HUVEC cells. KI67 is a cellular proliferation marker. The greater the KI67 labelling, the greater the gamma-H2AX background. This confirms the relation between the global level of gamma-H2AX and the cell-cycle phase of each cell. (A) Photograph of Texas Red®-X fluorescence associated with KI67 immunodetection. (B) Photograph of Alexa Fluor® 488 fluorescence associated with gamma-H2AX immunodetection. (C) Photograph of DAPI fluorescence associated with DNA content. (D) Merge.

**Fig 3:**
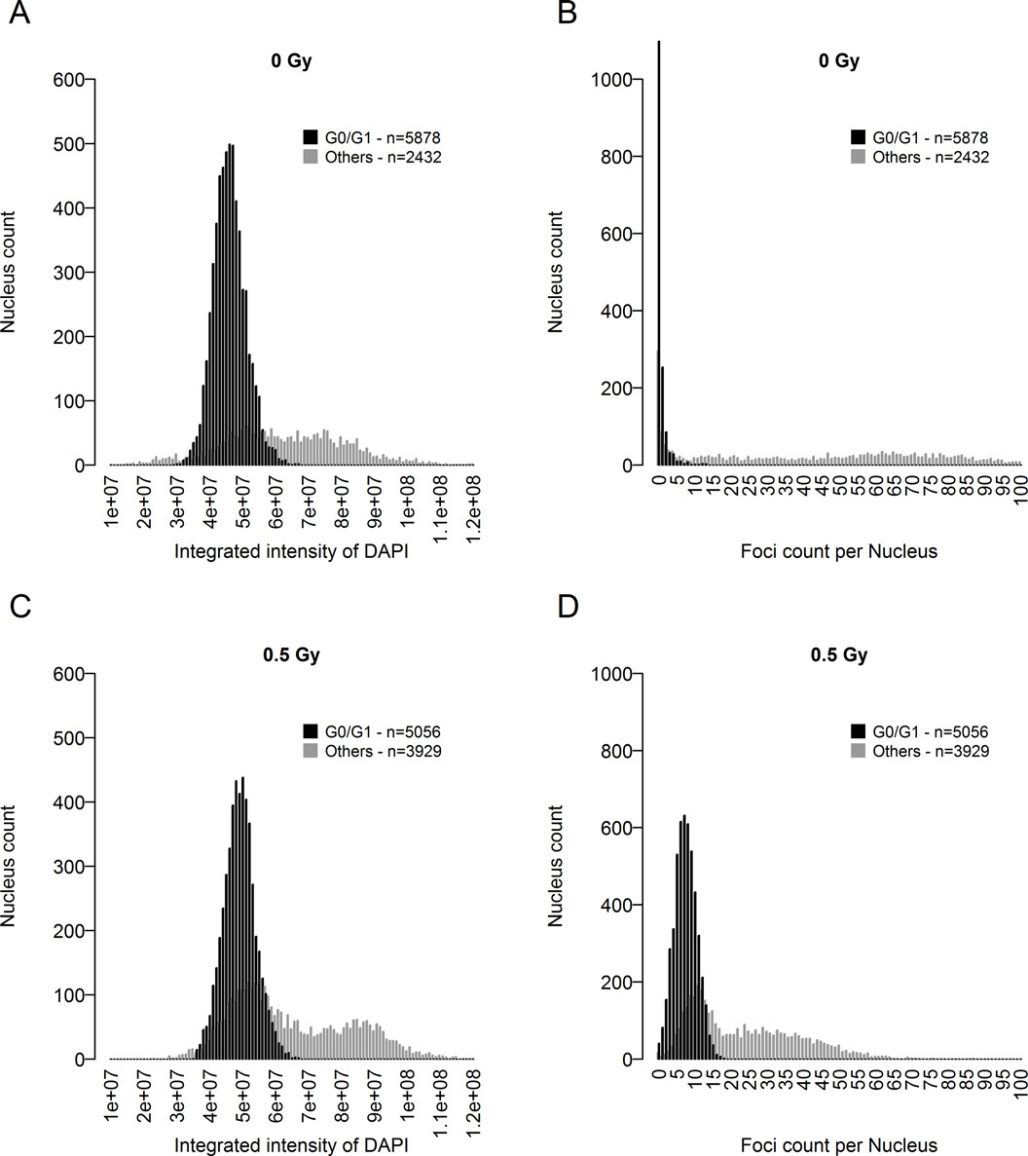
Histograms of nucleus count as a function of the integrated intensity of DAPI and number of gamma-H2AX foci. (A) and (C) correspond to histograms of the nucleus count as a function of their corresponding integrated DAPI intensities. (B) and (C) correspond to histograms of the nucleus count as a function of the corresponding number of gamma-H2AX foci. (A) and (B) Non-irradiated condition. (C) and (D) Irradiated condition, 0.5 Gy.

Another factor influencing the amount of DNA in target cells is the cell cycle. It was therefore necessary to irradiate a maximum of cells in the G0/G1 phase of the cell cycle. Moreover, cells undergoing division (S, G2 and metaphase) have a very high gamma-H2AX background. Thus, non-dividing cells must be selected to analyse gamma-H2AX foci that are specifically due to ionizing radiation. The most effective method was to produce cell cultures at high confluence and to be able to characterize the cell cycle state of each nucleus during image acquisition and data treatment. This characterization was based on the measurement of integrated intensities of DAPI, corresponding to the DNA content of each nucleus, and Alexa 488, corresponding to the gamma-H2AX level in the whole nucleus. This representation can be used to define a subpopulation of nuclei with a low level of integrated intensity of both DAPI and Alexa 488 in both non-irradiated (Fig 1A) and irradiated (Fig 1B) conditions. This allowed us to select a well-defined population of nuclei, mainly out of the cell cycle. Remarkably, the integrated intensity associated with the gamma-H2AX background due to cell division was affected very slightly, if at all, by the fluorescence associated with RIF at the doses tested (S1 Fig). As a control, we labelled KI67, a cell proliferation marker, to check the cell cycle state of the selected nuclei. As Fig 2 illustrates, the greater the KI67 labelling, the greater the gamma-H2AX background. On the contrary, non-irradiated nuclei with no or low KI67 detected also had no labelling for gamma-H2AX. This effect can be visualized at the scale of a cell population. Fig 3A and 3C plot the nucleus counts according to integrated DAPI intensity in a population of at least 1500 detected nuclei. The different phases of the cell cycle can be broadly distinguished in this representation and associated with the number of foci detected in the corresponding nuclei (Fig 3B and 3D respectively for 0 Gy and 0.5 Gy). Thus the unbiased selection of cells in the G0/G1 state is critical for studying the gamma-H2AX RIF.

The second step of cell characterization was to evaluate the typical nucleus size of HUVEC in the G0/G1 state and thus define a concrete volume to be used for calculating specific energy distribution and for validating the use of conventional microscopy for analysing RIF in a large population of several thousand nuclei.

**Fig 4:**
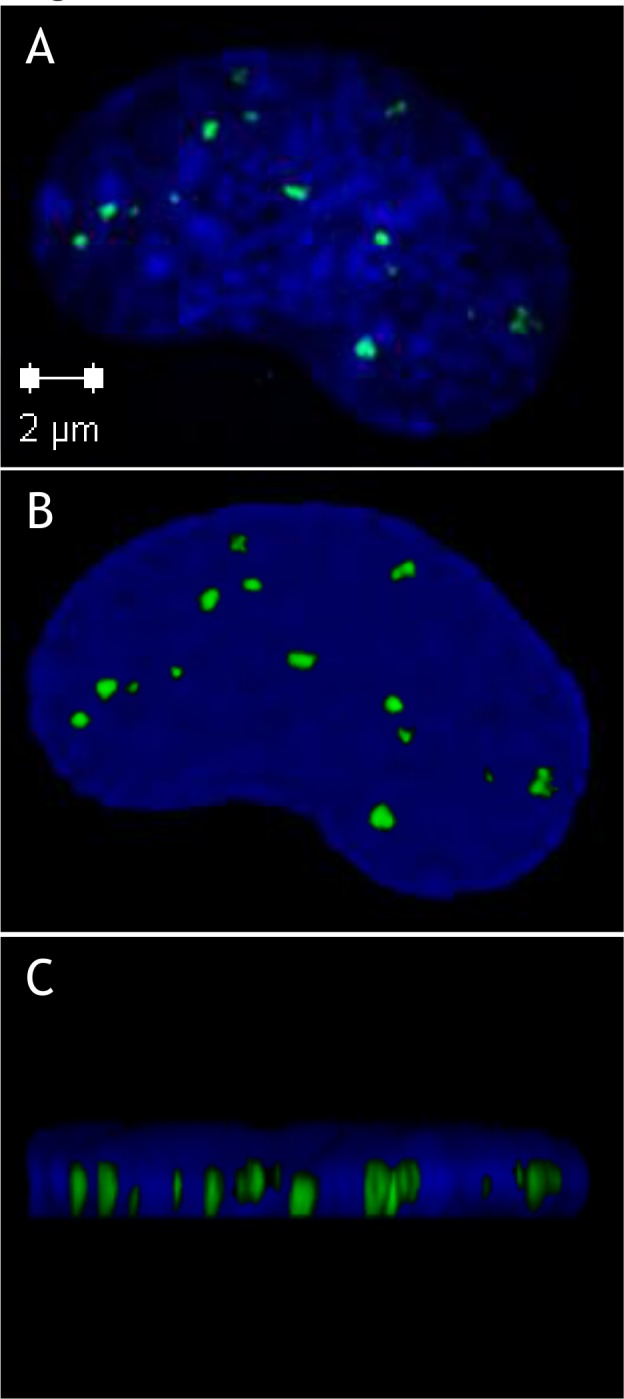
Labelling by immunofluorescence of gamma-H2AX acquired with confocal microscopy. This allows the study of the distribution of foci in the whole volume of irradiated endothelial cell nuclei, cultured as monolayers on glass in Lab-Tek^®^ chambers. (A) Gamma-H2AX foci viewed at the focal plane, as for conventional microscopy. (B) Top view after 3D reconstruction, with confocal microscopy acquisition of 15 layers across the nucleus. The number of foci is identical to that observed at the focal plane. (C) Side view of the 3D reconstruction. In view of the size of the foci, the thickness of the endothelial nucleus and the depth of field of the x63 objective used, it appears that all foci that can be detected with confocal microscopy can also be detected at focal plane with conventional microscopy. The picture is representative of all the nuclei examined by confocal microscopy.

For these purposes, we used confocal microscopy to measure the mean dimensions of the nuclei and to analyse the distribution of foci through the entire cell nucleus volume. Fig 4A shows gamma-H2AX foci viewed at the focal plane, as for conventional microscopy. Fig 4B and 4C show the foci for the same nucleus viewed after a 3D reconstruction with confocal microscopy. After confocal analysis of around 50 cells, the shape of nuclei was defined as an elliptic cylinder of dimensions 17±1.5 (major axis), 11±1.5 (minor axis) and 2±0.3 (thickness) μm ±SD. Given how thin the HUVEC nuclei are and the size of the RIF fluorescent volume (a sphere of 600 nm of diameter), it appears that all RIF detected with confocal microscopy can also be detected with a single focal plane with conventional microscopy. This statement is specific to our cells and culture conditions (monolayer on glass in Lab-Tek^®^ chambers). It does not appear applicable to other cell types with differently shaped nuclei, for which z-stacks are mandatory. For example, S2 Fig shows that it is not possible to detect all foci in lymphocyte nuclei with conventional microscopy and one focal plane.

### Dose Effect at the Macroscopic Scale

**Fig 5:**
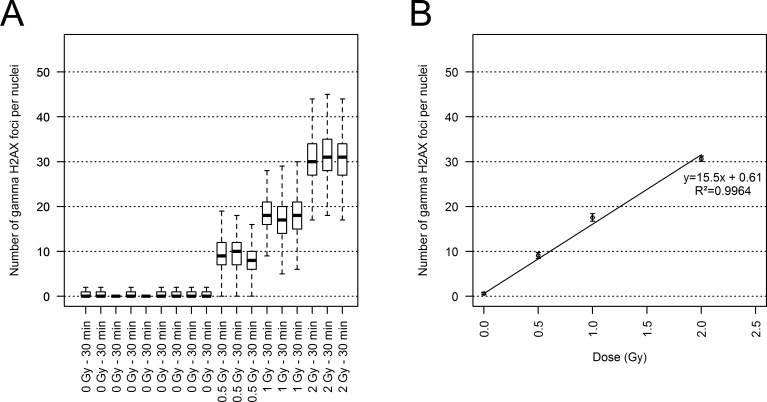
Dose-effect relations. (A) Box-and-whisker plot of the number of gamma H2AX foci per nucleus for each tested dose and their corresponding replications. Bold black bars of boxplots correspond to medians. The lower and upper borders of the box correspond to the first and third quartiles, respectively. The upper and lower whiskers correspond to 1.5 times the interquartile distance. (B) Linear regression based on mean values among replications performed for each tested dose. Error bars were calculated as the standard deviation between the average number of foci per nucleus obtained in each replication. Linear regression was done with the *lm* function of the *stats* package of R software.

HUVEC samples were irradiated with ^60^Co at two different macroscopic absorbed doses: 0.5, 1 and 2 Gy. Each experiment was independently replicated 3 times. Nine replicate experiments were performed for the non-irradiated condition. At least 1500 nuclei in G0/G1 were evaluated for each replication. The gamma-H2AX RIF were counted for each nucleus at 30 min after irradiation, because our preliminary analysis showed that this time point corresponded to the maximum RIF simultaneously observed per cell (data not shown). The boxplots in Fig 5A show the median RIF obtained for each condition and each replication. The mean RIF count per nucleus was 0.6±0.28 at 0 Gy, 9.1±0.66 at 0.5 Gy, 17.6±0.86 at 1 Gy and 30.7±0.60 at 2 Gy (Fig 5B). Errors were calculated as the standard deviation of the means obtained in each set of replications. These variations among replications appeared consistent with the uncertainty for the delivered dose, estimated at 6% for ^60^Co irradiation. A linear relation was obtained for the dose-effect curve, as shown in Fig 5B. From the slope of the linear relation we can estimate a mean RIF count per gray of 15.5±0.33.

### RIF Distribution among the Nuclei

Given the absence of differences among each set of replications, the variability in the RIF count for each population of nuclei was evaluated with pooled data. The final sizes of each population were 8242, 8993 and 9010 nuclei, respectively, for doses of 0.5, 1 and 2 Gy; the mean RIF counts were 8.8±3.2, 17.6±4.4 and 30.6±5.6, and both medians and modes were very close to the corresponding means (Table 1). Standard deviations (SD) were computed as the square root of the variance calculated for each population. This value allowed us to quantify the cell-to-cell variability in terms of the number of RIF. The relative standard deviations (SD^rel^, calculated as SD divided by the mean) for each dose population were 0.363, 0.253 and 0.182. Interestingly, the SD^rel^ was not constant and did not increase with the dose: the SD increased with dose, but the SD^rel^ decreased. The dose effect relation appeared to be linear: when the dose was multiplied by 2, the mean number of RIF per nucleus also doubled, but the corresponding SD did not. These results were confirmed with independent irradiations with two different X-ray sources. At each dose, the various parameters of the distributions were around the same, regardless of the type of radiation. More specifically, the variance of the distribution of the number of RIF per nucleus in the cell population appeared to be equal to the mean of the distribution. Table 1 summarizes all these descriptive statistics.

**Table 1:** Observed values for RIF.

Source	Dose^a^	nucleus number	Mean^b^	Median^b^	Mode^b^	Var	SD	SD^rel^	Fold change SD^rel^
	0	25114	0.6	0	0	2.1	1.5	2.558	
**Cobalt 60**	0.5	8242	8.8	9	8	10.2	3.2	0.363	0.696
	1	8993	17.6	18	17	19.7	4.4	0.253	
									0.719
	2	9010	30.6	31	31	30.9	5.6	0.182	
**X rays 4 MV**	0.5	7362	10.2	10	11	10.7	3.3	0.322	0.732
	1	8721	17.3	17	16	16.7	4.1	0.236	
									0.785
	2	5162	33.2	33	34	38.0	6.2	0.185	
**X rays 200 kV**	0.5	1555	8.8	9	9	9.81	3.1	0.357	0.710
	1	6796	17.6	18	18	19.76	4.4	0.253	
									0.759
	2	8149	30.2	30	31	33.73	5.8	0.192	

^a^ gray

^b^ Radiation induced foci per nucleus

### Distribution of Specific Energies Calculated in Nuclear and DNA Volumes

The main objective of these calculations was to obtain an evaluation of the dispersion of the energy deposition in the different target volumes and subvolumes in which RIF can be generated. Eq 3 was therefore used to calculate the relative standard deviations of the specific energy distributions for the ^60^Co irradiations. Table 2 presents the descriptive statistics obtained for each of the absorbed doses in the biological experiments (0.5 Gy, 1 Gy and 2 Gy). These statistics were calculated from data generated assuming a normal distribution for the same number of events as in the biological experiments. Initially, two different target volumes were studied: 293.7 μm^3^, the volume of the whole cell nucleus, and 8.5 μm^3^, the volume of a 6-Gbp DNA molecule within the nucleus. The first volume was calculated from dimensions determined by the biological experiments, and the second on the assumption that a DNA molecule of 6 Gbp is a cylinder with a diameter of 2.3 nm and a height of 2.04 m. As expected, the standard deviation of the specific energy distribution within the population of targets increased as the target size decreased. For the DNA molecule, for example, the variance increased approximately 20-fold. Interestingly, for each of these two volumes, SD increased with dose while SD^rel^ decreased. It is important to note that both these volumes were calculated from mean sizes that were measured or estimated. Uncertainties and variability among the nuclei of each cell population are associated with these sizes. For a given target, however, a variation of about 15% around the mean volume did not significantly change the spread of the specific energy distributions that we calculated. Accordingly, considering this kind of variability for calculating these spreads was unnecessary because it did not significantly change the results of the calculation.

**Table 2:** Calculated values for specific energy.

Target volume	Dose^a^	Number of synthetic objects	Mean^a^	Median^a^	Var	SD	SD^rel^	Fold change SD^rel^
**Calculation for whole nucleus (293.7 µm3)**	0.5	8242	0.500	0.501	0.00047	0.022	0.043	0.708
	1	8993	1.000	1.001	0.00095	0.031	0.031	
								0.707
	2	9010	2.000	2.001	0.00190	0.044	0.022	
**Calculation for DNA molecule (8.5 µm3)**	0.5	8242	0.501	0.502	0.00867	0.093	0.186	0.709
	1	8993	1.001	1.003	0.01736	0.132	0.132	
								0.707
	2	9010	2.002	2.004	0.03473	0.186	0.093	
**Calculation for a target volume of 1.6 µm3**	0.5	8242	0.503	0.504	0.03400	0.184	0.367	0.710
	1	8993	1.002	1.005	0.06814	0.261	0.260	
								0.708
	2	9010	2.003	2.007	0.13627	0.369	0.184	

^a^ gray

### Comparison between Distributions obtained for Specific Energy and for RIF

**Fig 6:**
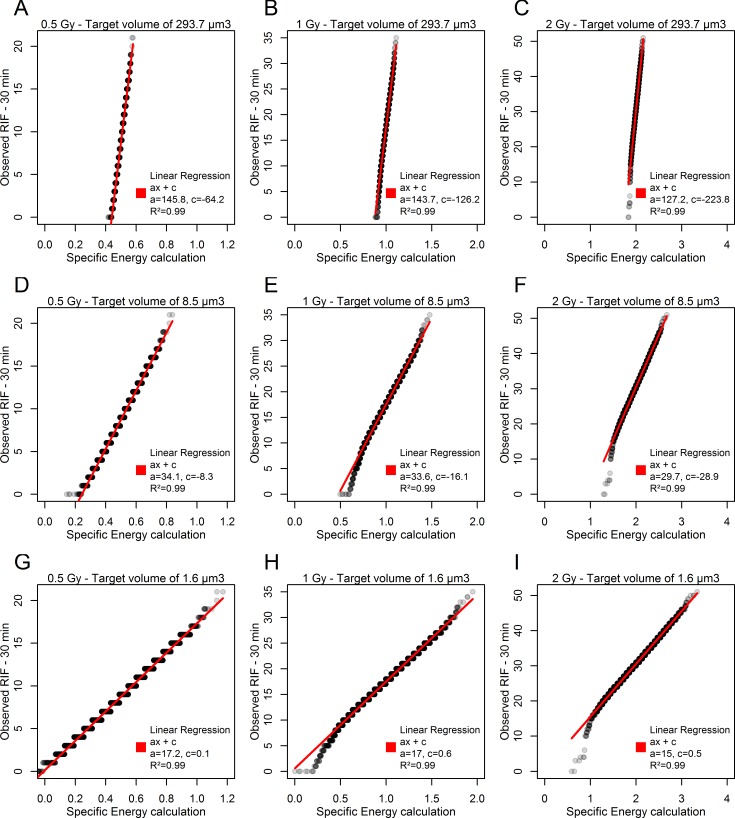
Q-Q plots for comparing probability distributions from experiments and simulations by plotting their quantiles against each other. Linear regression was done with the lm function of the stats package of R software. (A), (B), and (C) correspond to Q−Q plot of observation versus simulation for doses of 0.5, 1 and 2 Gy respectively considering a target volume of 293.7 μm^3^. (D), (E), and (F) correspond to Q−Q plot of observation versus simulation for the same doses, considering a target volume of 8.5 μm^3^. (G), (H), and (I) correspond to Q−Q plot of observation versus simulation for the same doses, considering a target volume of 1.6 μm^3^.

The results obtained experimentally and via specific energy calculation cannot be compared directly as they do not represent the same quantity (RIF per nucleus for the first, and Gy per target for the second). Nonetheless, some comparison can be made with the relative standard deviations for each (Table 1 and 2) as well as with Q-Q plots (Fig 6).

**Fig 7:**
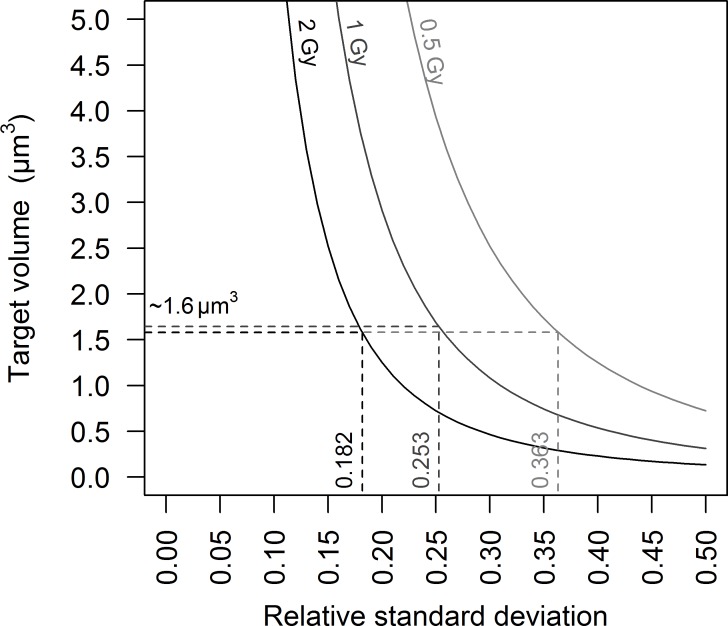
Graphic representation of the relation between the SD^rel^ of the specific energy distribution and the target volume. The relations were plotted with Eq 3, using k_1_ and k_2_ calculated by [15] for ^60^Co. The relations for doses of 0.5, 1 and 2 Gy are represented by light grey, grey and dark grey curves respectively. A target volume of around 1.6 μm^3^ was calculated to explain the totality of the SD^rel^ of the RIF distribution by the spread of energy specific distribution, regardless of the dose considered.

The SD^rel^ were not constant with dose changes for either RIF or specific energy distributions. SD increased with dose while SD^rel^ decreased with dose, and the rates of increase and decrease were approximately the same for the biological and physical measurements. For example, when the mean doubled between the doses 0.5 and 1 Gy, the corresponding fold change for the specific energy calculations and the RIF observation were the same: 1.4 for the SD and 0.7 for the SD^rel^. This was observed for all irradiation sources tested and all target volumes considered. Nonetheless, the SD^rel^ of the specific energy distribution remained smaller than the SD^rel^ of the observed distribution of the number of RIF per nucleus: the latter was twice as large as the first for a volume corresponding to DNA molecule (8.5 μm^3^) and around 8 times larger than the first for the whole nucleus (293.7 μm^3^). Using Eq 3 and for ^60^Co, we calculated a third target volume that might produce a SD^rel^ for specific energy similar to that observed for RIF. Hence for the SD^rel^ of 0.363, 0.253 and 0.182 (corresponding to 0.5, 1 and 2 Gy respectively) (Table 1), we calculated similar target volumes around 1.6 μm^3^ (Fig 7).

The Q-Q plot method is used for comparing probability distributions from experiments and simulations by plotting their quantiles against each other. These probability plots and the associated linear regression analysis appear to show a good match between the quantiles obtained by calculations and those obtained by observation of RIF. Fig 6G, 6H and 6I show the Q-Q plots obtained for the smallest target volume (1.6 μm^3^). This volume produces the same SD^rel^ for the RIF and specific energy distributions at each dose tested. Note that, in this case, the slope of the regression line obtained for each dose corresponds to the observed mean number of RIF per gray.

## Discussion

This study accurately estimated the distribution of the number of RIF per cell in a large population of primary and “low passage” endothelial cells exposed to ^60^Co. To be able to interpret the intercellular variability of RIF count per cell and be certain that intercellular variations in the initial DNA content were not the source of this post-exposure RIF variability, we developed an approach that combines the advantages of flow cytometry and microscopy. We demonstrated that this approach can sort a cell population according to the state of the cell cycle, as flow cytometry can. It also allows the accurate analysis of the number of RIF in each cell of a given subpopulation.

Other studies have used flow cytometry to investigate the level of DNA damage in large cell populations [16–19]; this technique can be used to measure the intensity of fluorescently tagged biomolecules within individual cells. By staining cells with propidium iodine or DAPI and a fluorescently labelled antibody conjugated to a DNA damage response phospho-protein, several authors of flow cytometric studies have been able to determine both the cell cycle phase and the level of specific phospho-proteins in individual cells within a population [19]. This methodology has been successfully used to compare the effect of low LET and high LET radiation (photon vs Iron-Ion Exposure) and enabled the study of how radiation quality and cell cycle stage affect phospho-kinetics [17]. However, flow cytometry measures an absolute intensity of gamma H2AX antibody binding per cell. Although the dose–response relation between mean total gamma H2AX fluorescence levels and mean RIF numbers is similar after low-LET radiation exposures, the distributions of phosphorylation levels are strongly skewed [16,17]. Despite its simplicity and speed, this methodology does not appear sensitive enough to characterize accurately the intercellular RIF variability for a given dose of photon radiation. The methodology used in our study enabled us to estimate the RIF count variance accurately among a large population of cells exposed to ionizing radiation and thus to observe and quantify significant cell-to-cell variability in the number of gamma H2AX RIF. This observation is especially important for photon radiation because it underlies the likely variation in cell response and behaviour. Cell fate is indeed linked to its rate of DNA-induced damage. The analysis of the distribution of RIF per nucleus within the population of cells exposed to 1 Gy of ^60^Co gamma rays shows that 15% of the cell nuclei had from around 25% (the 85th centile of the distribution) to almost 200% (maximum of the distribution) more RIF than the median of the population. On the other hand, for a fraction used in standard radiation therapy (*i*.*e*., 2 Gy), the DNA damage rate for around 15% of the cell population is 18% lower than the mean rate. Moreover, the damage rate for around 1% of the cells exposed to 2 Gy is lower than the mean rate observed for 1 Gy. This result should be taken into account in cancer cell treatment, where the survival of a few cells may be the source of relapse. This kind of heterogeneity is well characterized for high-LET radiations, because of the condensed distribution of the energy deposits along the particle track. It has been studied and described much less frequently for such low-LET radiation as photons. Most studies measuring modulations in, for example, gene expression or protein level in response to ionizing radiation must work at the level of whole cell population because minimum amounts of material are required for measurements. When a significant modulation is observed, two contrary explanations are possible: all the cells modulate expression around the measured mean value or only a subpopulation of cells modulates expression to a much higher level than the average value measured. When modulation is dose dependent, these two hypotheses lead to distinct conclusions: modification of the expression level in each cell with the dose, or change as a function of dose in the proportion of the subpopulation which responds. For high LET particles, when the fluency decreases, the probability that key components of a cell will be damaged decreases, so that the second alternative is most likely. In the case of photon radiation, the first alternative is generally preferred. However, our results show clearly that for this kind of exposure all cells do not signal the same amount of damage and that the variation among cells is far from negligible. Given that signalling DNA damage is one of the first steps in cellular response to ionizing radiation insults, our observation may imply a significant variation in each individual cell response, especially if there are threshold effects for some cellular responses. Moreover, this relative cell-to-cell variability increased as the dose decreased, contrary to the mean number of RIF per cell. This result underlines that the dose effect, in terms of the number of DNA lesions indicated by RIF is not as simple as a purely proportional relation in which relative SD is constant with dose.

For these reasons, it is interesting to analyse the different potential origins of the cell-to-cell variability we observed. Several parameters can contribute to the measured standard deviation of the number of RIF per cell within the cell population. Three sources can be identified: measurement errors, biological variability and the stochastic nature of energy deposition.

**Fig 8:**
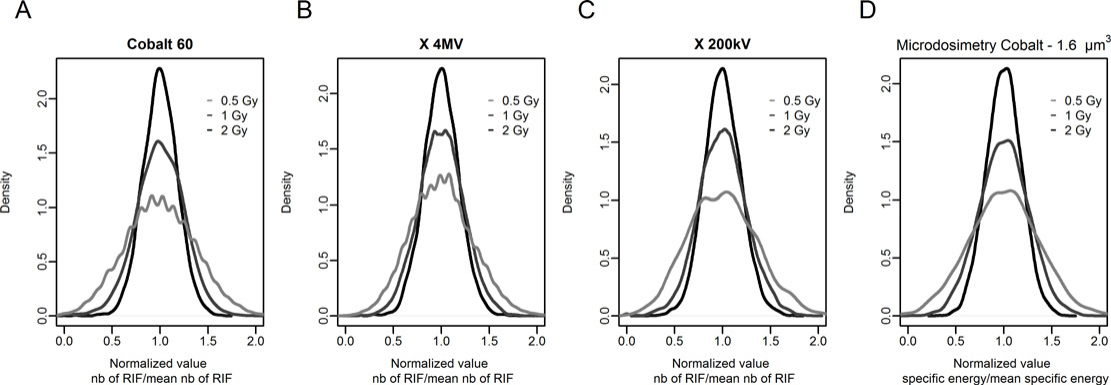
Density plot of normalized RIF count per nucleus and normalized computed specific energy per volume of 1.6 μm^3^. (A), (B) and (C) Density plot of normalized RIF count per nucleus measured after exposure of cells to macroscopic doses of 0.5 (light grey line), 1 (dark grey line) and 2 Gy (black line) for 3 different sources of radiation: ^60^Co, 4 MVp X-rays and 200 kVp X-ray, respectively. (D) Density plot corresponding to the distribution of normalized computed specific energy per volume of 1.6 μm^3^. This was calculated from data generated with Eq 3, an assumption of a normal distribution, and the same number of events as in the ^60^Co biological experiments. The light grey, dark grey and black lines correspond to macroscopic doses of 0.5, 1 and 2 Gy respectively.

First, the error related to the measurements and a portion of the biological variability can be estimated from the standard error calculated with the different replications performed for each dose of ^60^Co radiation (Fig 5B): 0.28 for 0 Gy, 0.67 for 0.5 Gy, 0.86 for 1 Gy and 0.60 for 2 Gy. These measured errors are significantly smaller than the standard deviations measured in the same population of nuclei (3.2, 4.4 and 5.6 for 0.5 Gy, 1 Gy and 2 Gy respectively, as shown in Table 1) and are consistent with the estimated uncertainty for the delivered dose for ^60^Co irradiation: 6%. At the same time, no correlation seems to exist between the errors measured and the doses studied. These errors would therefore not contribute to the relation observed between doses and standard deviations calculated from the distributions of the number of RIF per cells (Table 1 and Fig 8A, 8B and 8C).

Second, regarding the biological variability, the kinetics of signalling and repairing DNA damage is a critical parameter that may play a role in the dispersion of these RIF per nucleus distributions. As the work of Neumaier et al [20] illustrates, signalling and repair are dynamic processes that have, *a priori*, no reason to be perfectly synchronized from one cell to another, even in a population of cells simultaneously exposed to radiation. Thus, a portion of the cells with a low number of RIF could correspond to cells that have not yet completely signalled all their DNA damage or that have already repaired some of it. In Fig 6, we observe, for example, a deviation from the linear regression specifically for the nuclei with the fewest RIF per nucleus. This curvature corresponds to cells with fewer RIF detected than expected according to the linear relation between specific energy and RIF per nucleus. This deviation may thus illustrate the presence of cells asynchronous in signalling DNA damage.

The third phenomenon potentially involved in the variation observed in the number of RIF per nucleus is the stochastic nature of energy deposition. This number reflects a quantity of damage, generally assessed as DSBs, to the DNA molecule. At the scale of DNA, the initial number and position of DSBs should depend on the energy deposition structure at a micrometric scale—at which the energy deposition for photon radiation is not uniform [21]. For equivalent small volumes at the micrometric scale (target volumes and subvolumes in which RIF can be generated), the spread of the energy deposited by ionizing radiation is due to fluctuations in both the number of tracks passing the target and the energy deposition per track [11–14]. Thus the energy absorbed by a specific target follows a frequency distribution f(z) where z is a stochastic quantity corresponding to ε, the energy imparted per mass m, in the target of volume V. The macroscopic dose D corresponds to the expected value of the distribution f(z). Following F. Villegas et al. [22], we calculated variances, standard deviations and relative standard deviations for 3 target volumes and 3 doses of ^60^Co and we observed that these values changed with the dose in the same way for the calculations and the observations (Tables 1 and 2, Fig 8). The similarity of this behaviour is an important argument in supporting the hypothesis of the conservation of the link between the two phenomena: number of RIF per nucleus and specific energy absorbed per target volume. More specifically, it appears that when the macroscopic dose doubles, the relative standard deviation is changed by a factor of around $\frac{1}{\surd 2}$, both for RIF and calculated specific energy. This relation is independent of the target volume considered. The same relation was observed for the distribution of the number of RIF per nucleus for the other two radiation qualities tested (4 MVp or 200 kVp X-ray). This relation between mean and relative standard deviation is typical of distributions for which variance is equal to the mean, such as Poisson’s distribution. Our data show that this relation was more or less present in all distributions of RIF counts per nucleus (Table 1). It is also accepted that there is a Poisson component in the calculation of the distribution of specific energy in a given volume, associated with the fluctuation of the number of electron tracks passing through the volume [11–14]. Consistent with these observations, we hypothesise that the number of DSBs on a target is associated with the number of electron tracks through the same target.

The volume of the target is interesting to analyse. If we consider the total volume of the endothelial cell nucleus as the target volume (293.7 μm^3^), the variation of specific energy absorbed from one nucleus to another explains roughly 10% of the SD^rel^ measured for RIF. In this case, around 90% of the variation in the number of RIF from one nucleus to another could be attributed to measurement error and biological variability, as explained above. This assumption does not appear likely, given that the RIF reflect damage to a specific molecule, DNA, which occupies less than 3% of the volume of the nuclei of the endothelial cells we used. The total nuclear volume thus does not appear to be a relevant target volume. Energy deposition explains around 50% of the RIF count variation between nuclei when we consider the volume of the hydrated DNA molecule—8.5 μm^3^ (including hydration shell water). We calculated that a target volume of 1.6 μm^3^ would explain the totality of the SD^rel^ of the RIF distribution by the spread of specific energy distribution (Fig 7). This value is consistent with the order of magnitude of the volume occupied by the hydrated sugar-phosphate backbone of the DNA molecule, which is the part of the DNA molecule responsible for strand breaks. Moreover, following up this hypothesis, we observe a linear relation between the number of RIF per nucleus and the specific energy absorbed per sugar-phosphate backbone, regardless of the macroscopic dose (Fig 6G, 6H and 6I). It is important to note that the constants and slopes calculated from these different relations are consistent with the foci background and number of RIF per gray calculated in Fig 5 and measured in Table 1.

Although this hypothesis is not very intuitive, considering that the variance of observed RIF is completely explained by the variance of the specific energy distribution in the volume of the sugar-phosphate backbone of the DNA molecule might be consistent with the existence of indirect effects. This would require that the amount of scavengeable damage, as defined by Ward [23], is a constant percentage over the cell population of the amount of unscavengeable damage (also improperly called direct damage) produced in a given cell. In this case, the value of SD^rel^ of the distribution of the number of RIF per nucleus would be determined by the mean and variance of unscavengeable damage or lesions, which appear to be linked to the mean and variance of specific energy in a volume of 1.6 μm^3^. Our laboratory is currently investigating the SD^rel^ of the number of RIF per nucleus in the presence of scavengers of reactive oxygen species.

An alternative hypothesis can also be applied to the result we obtained with our assumption of a target volume of 1.6 μm^3^. Recent work by Lorat *et al* [24,25] suggests that that the RIF observed at 30 min after exposure by immunofluorescence detection of 53BP1 or gamma-H2AX could correspond to damage that occurs in a specific region of the chromatin. After using transmission electron microscopy to locate gold-labelled pKu70, 53BP1 and gamma-H2AX, they described different kinetics of resolution of DNA damage and different signalling partners, all varying according to the location of the damage. In sparse DNA regions (defined as euchromatin by the authors), they observed fast signalling kinetics (maximum at 5 min post-irradiation), with pKu70 dimers and neither 53BP1 nor phosphorylation of H2AX. In dense DNA regions (which they defined as heterochromatin), they described slower signalling kinetics (maximum at 30 min post-irradiation), with pKu dimers associated with 53BP1 or phosphorylation of H2AX. Although they did not control for the cell cycle state of the analysed cells, their results may suggest that the distribution of RIF number per nucleus observed in our study corresponds to damage occurring in dense DNA regions. Thus, the target volume of 1.6 μm^3^ could reflect the volume of DNA in that specific state in our cells. Additionally, this assumption leads to two non-exclusive consequences. First, if the percentage of condensed DNA is constant in a population of a given cell type, then for a given dose and radiation quality, it should play a role in the variation of the mean and variance of the RIF distributions between two cell types showing a significant difference in that percentage. Second, the variability of the heterochromatin/euchromatin ratio among cells of a given cell type might also contribute to the variance of the RIF distribution. These two points are currently under investigation.

Microdosimetry showed that, for a given macroscopic absorbed dose, variations of microscopic specific energy could be measured in volumes of the order of magnitude of key molecules in the cell, such as the DNA molecule, even for photon irradiation [11–14,21,26]. As far as we know, our study is the first to report a putative biological consequence of this characteristic of energy deposition due to photon exposure at a submicrometric scale. The study of the relation between the fraction of cells with a rate of DNA damage significantly higher than the median of the population and their behaviour appears interesting. The reasons that only a fraction of cells die, for example, at a given macroscopic dose are not well understood. A combination of the variability of the initial biological state of cells and the stochastic production of critical damage has been generally hypothesized to explain the fate of a portion of the exposed cell population. However, in light of our results, it seems particularly interesting to study the contribution of the spatial distribution of energy deposition at the scale of key cell molecules, such as DNA, even for photon exposure. Preliminary Monte Carlo calculations using low energy photons show that the variance of absorbed dose in a population of DNA molecules is greater than that calculated for ^60^Co irradiation (data not shown). Biological confirmation of the results of these simulations with low energy X-rays should be investigated.

In conclusion, this study demonstrates how biological observations and specific energy calculation can converge to improve understanding of the consequences of irradiation at the scale of a key cell component, such as DNA. The results presented here, based on the convergence of two approaches, could help to improve the evaluation of risk associated with the different types of radiation qualities. Heterogeneity of the response at the cell level might have consequences for the more integrated response of cell populations. We demonstrate that microdosimetric quantities might predict this heterogeneity. These quantities might then be usefully considered in several fields of radiation biology, including studies of non-targeted effects and low dose effects but also for clinical applications, such as improving treatment planning or evaluating or identifying adjuvants that might improve therapeutic effectiveness by increasing the homogeneity of energy deposition at the micrometric scale—the scale of the target molecules.

## Supporting Information

S1 Fig Scatter plots of the integrated intensities of DAPI and Alexa Fluor® 488 for each replication and all conditions tested. The integrated intensities of DAPI and Alexa Fluor® 488 correspond respectively to the DNA content and level of gamma-H2AX in the whole nucleus of each analysed cell. Green dots indicate a well-defined population of nuclei mainly in the G0/G1 phase of the cell cycle used to performe the RIF analysis. Red dots correspond to nuclei not used for the RIF analysis. (A) to (R): the green subpopulation could be easily discriminated in all conditions tested.
(PDF)

S2 Fig Labelling by immunofluorescence of gamma-H2AX acquired with confocal microscopy in lymphocyte. This allows the study of the distribution of foci in the whole volume of irradiated lymphocyte nuclei. (A) Gamma-H2AX foci viewed at the focal plane, as for conventional microscopy. (B) Top view after 3D reconstruction, with confocal microscopy acquisition of 45 layers across the nucleus. The number of foci is higher to that observed at the focal plane. (C) Side view of the 3D reconstruction. In view of the size of the foci, the thickness of the lymphocyte and the depth of field of the x63 objective used, it appears that all foci that can be detected with confocal microscopy cannot be detected at focal plane with conventional microscopy. The picture is representative of all the nuclei examined by confocal microscopy.
(TIF)
