# Supplementary figures and images for: Cell to Cell Variability of Radiation-Induced Foci: Relation between Observed Damage and Energy Deposition
Source: PLoS One. 2016 Jan 4;11(1):e0145786. doi: 10.1371/journal.pone.0145786 (PMC4699766; doi:10.1371/journal.pone.0145786)

# supplemental Figure 1

A

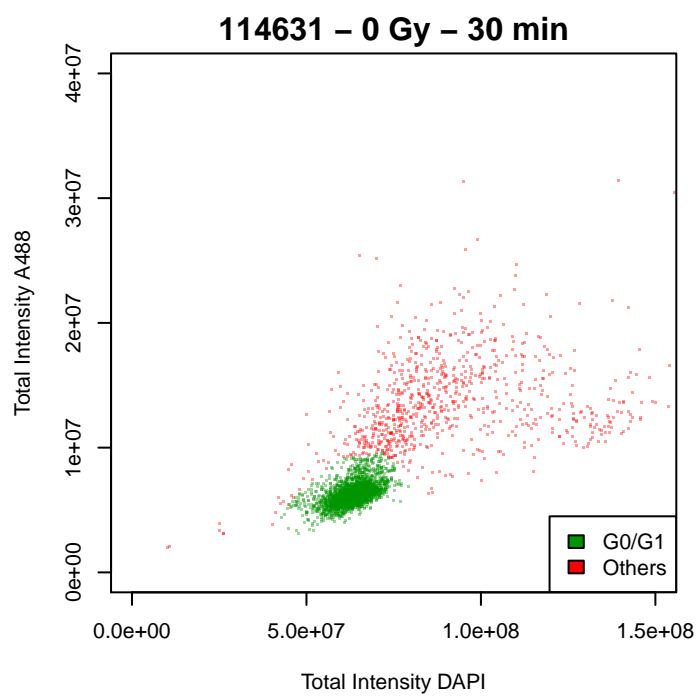

B

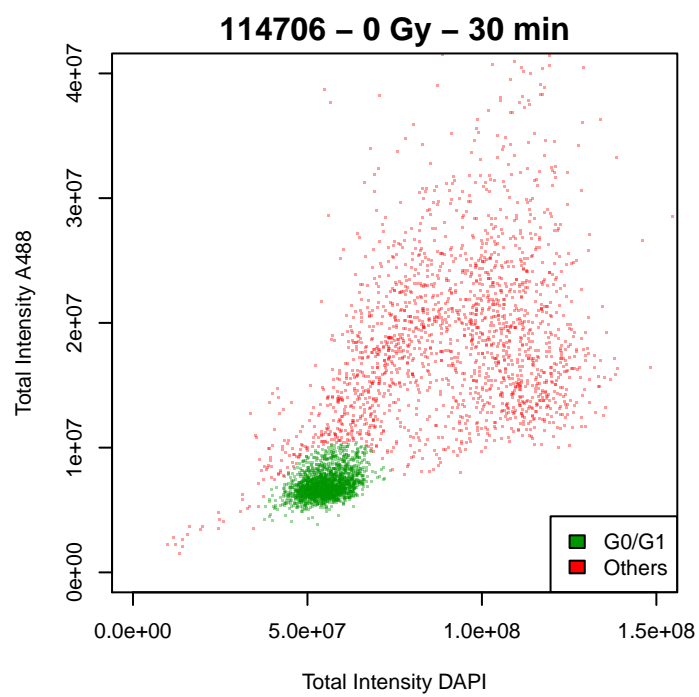

C

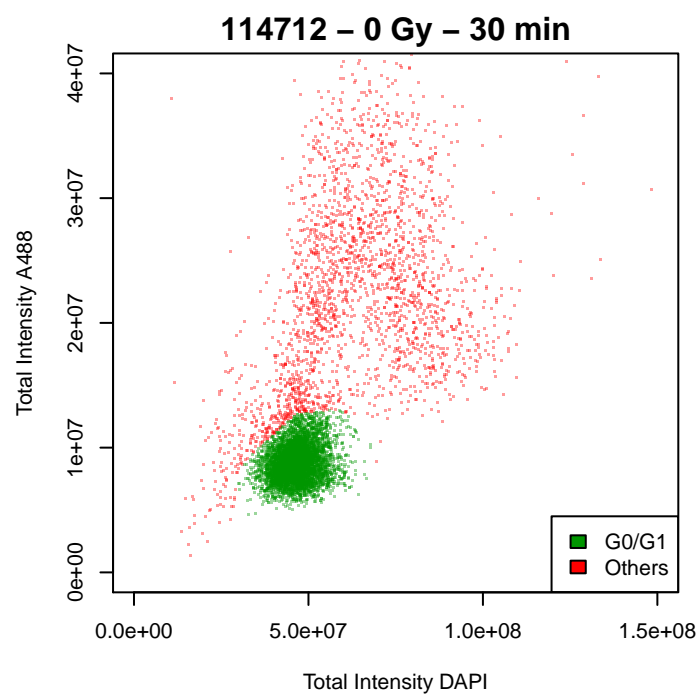

D

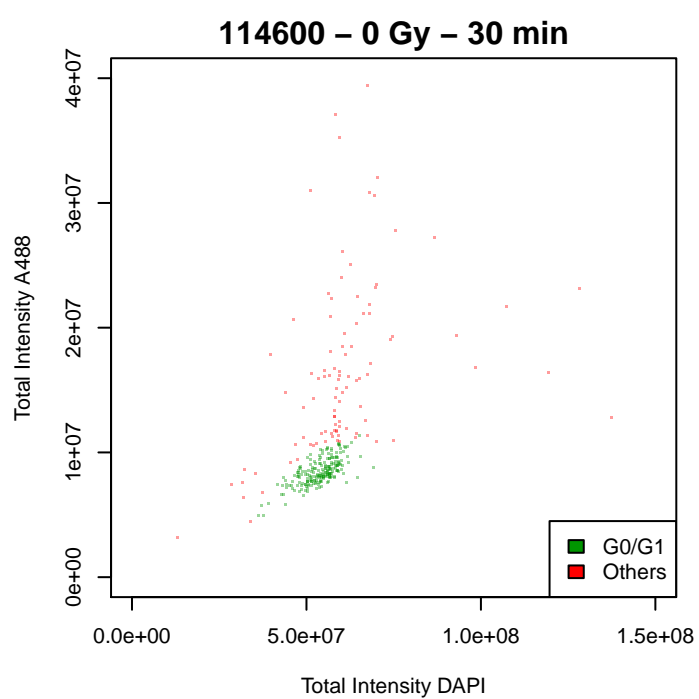

E

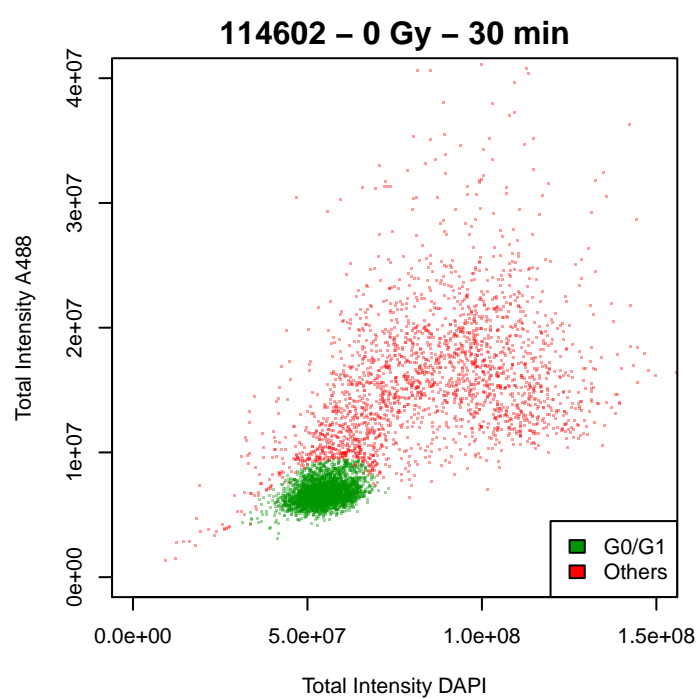

F

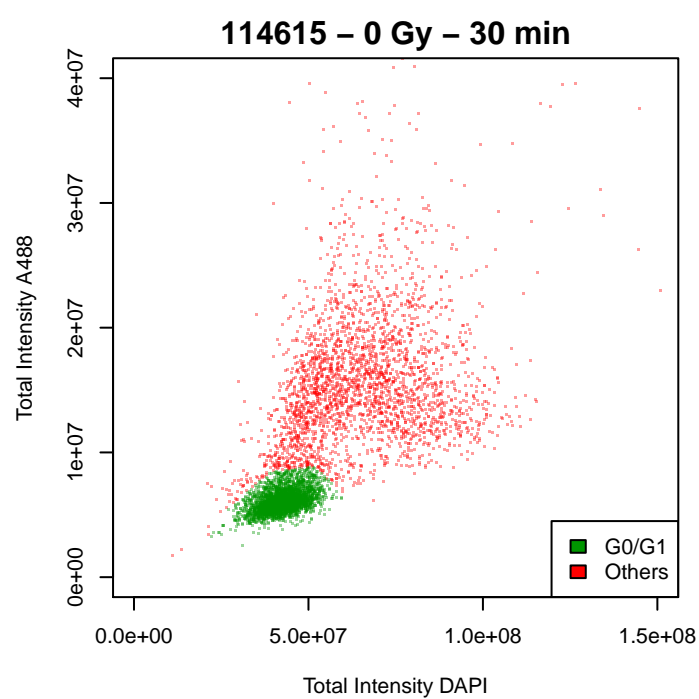

G

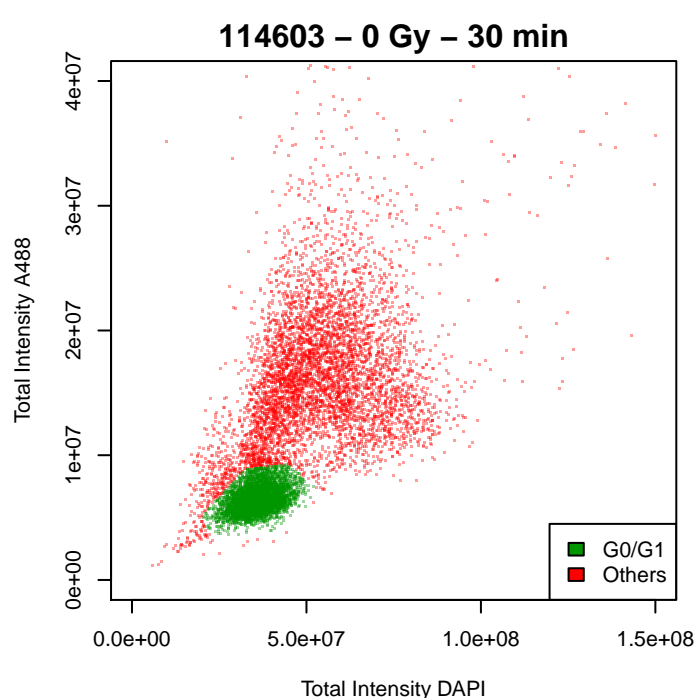

H

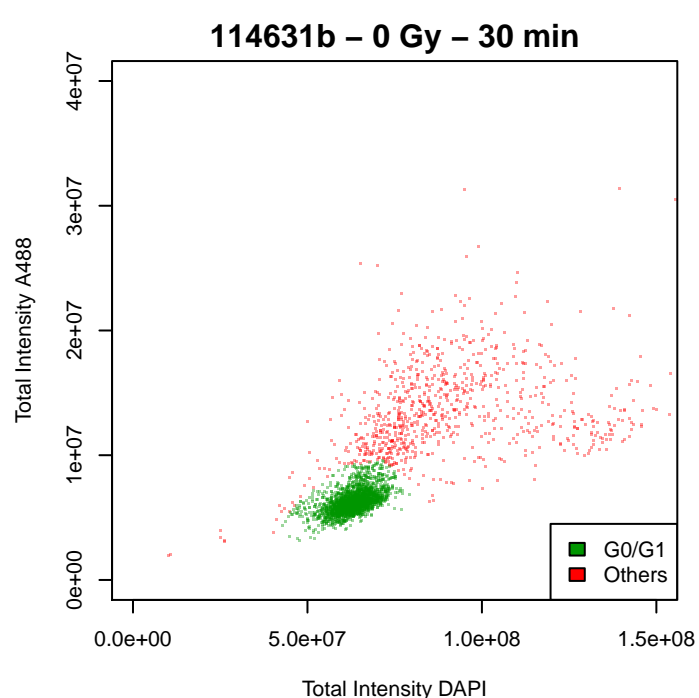

I

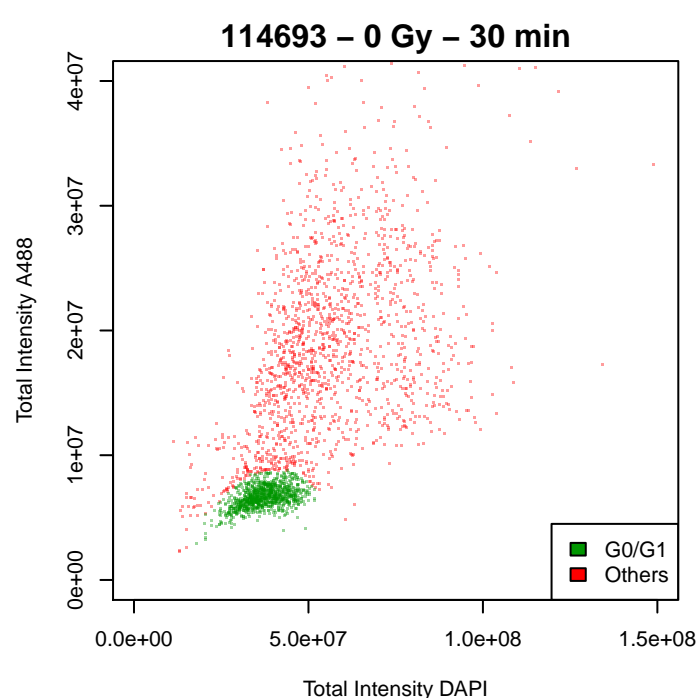

J

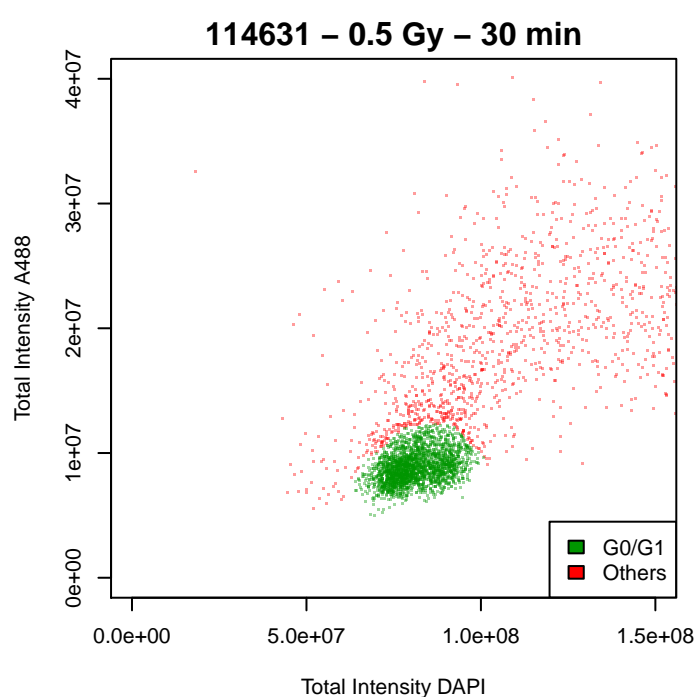

K

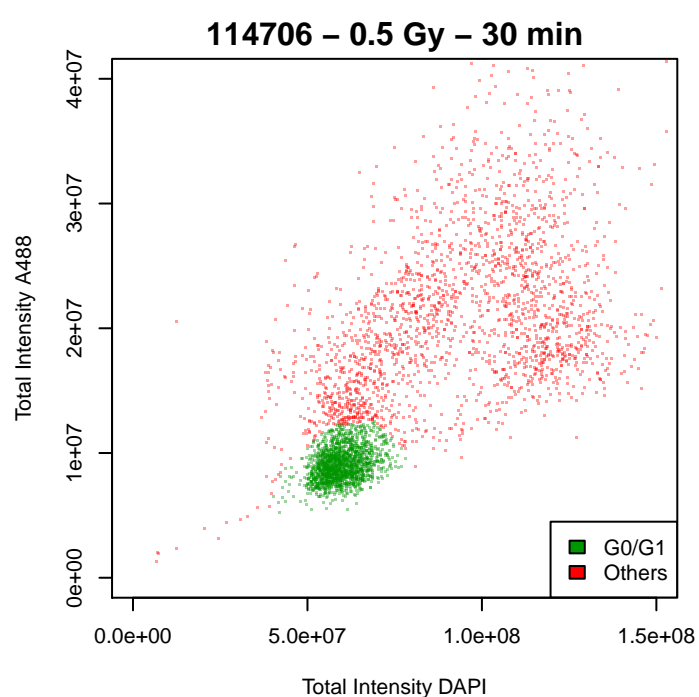

L

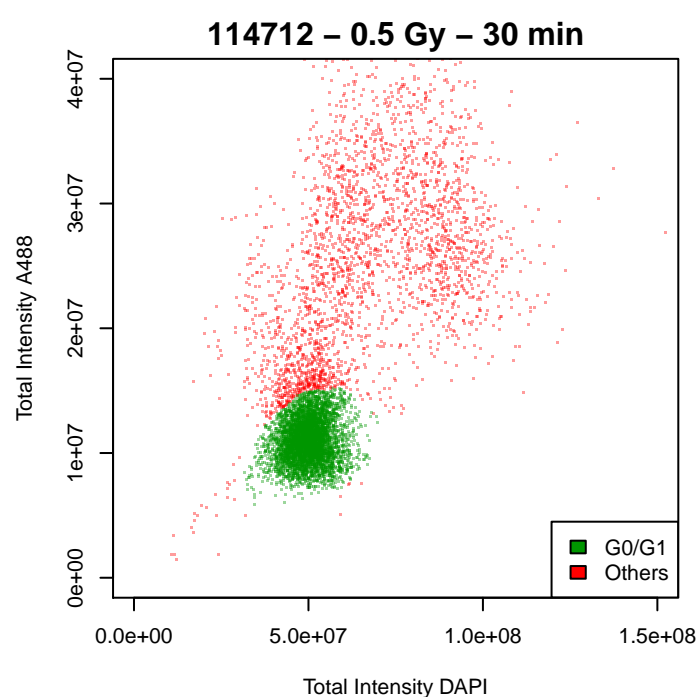

M

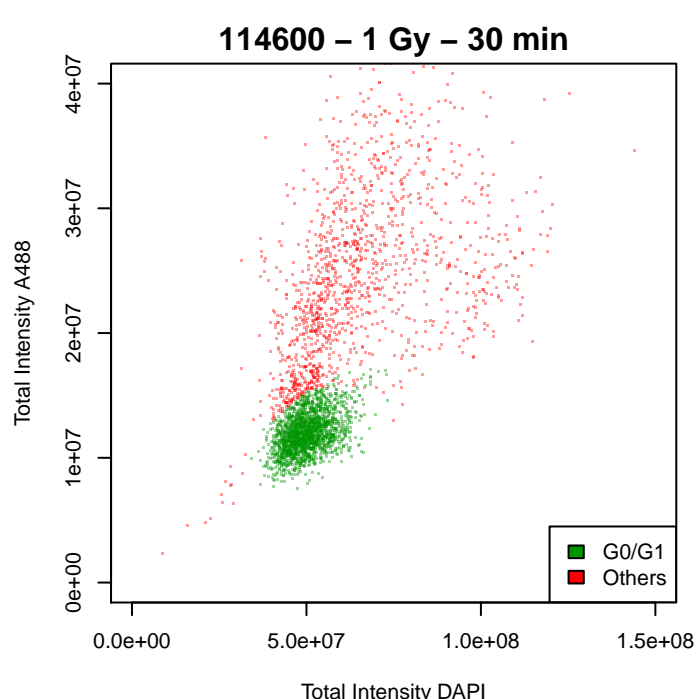

N

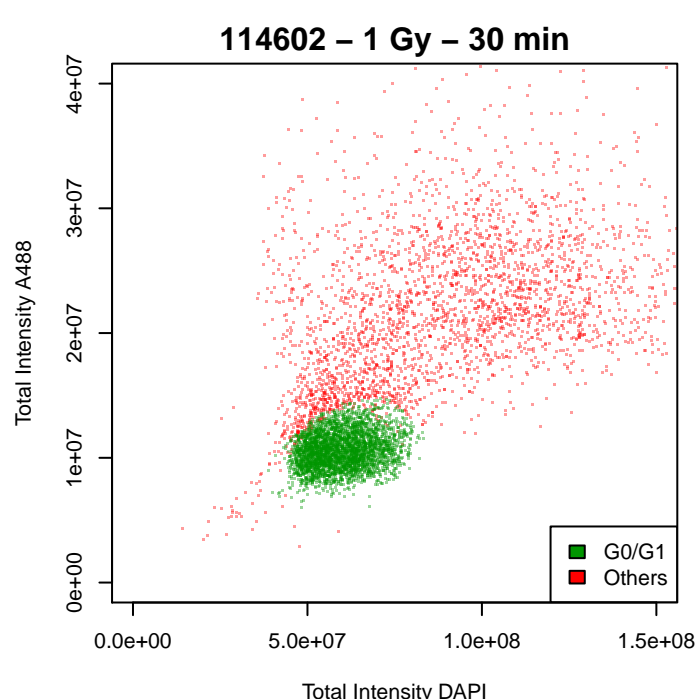

O

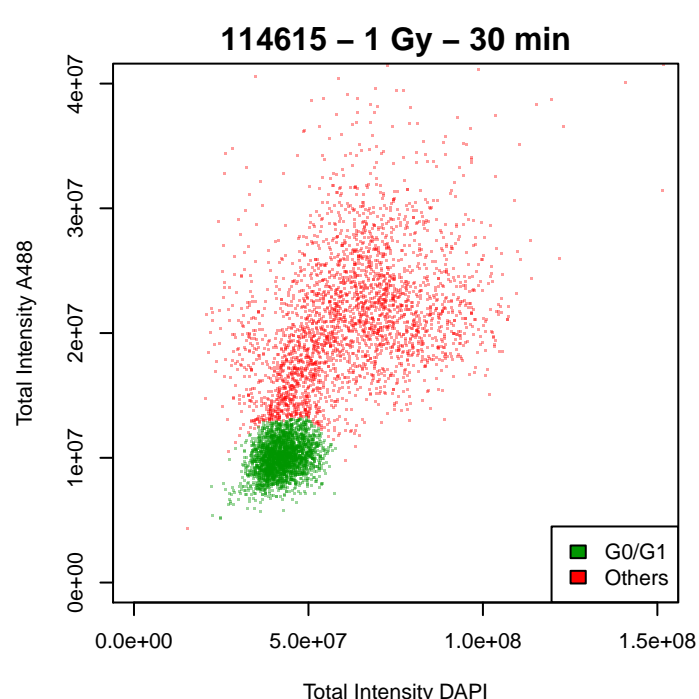

P

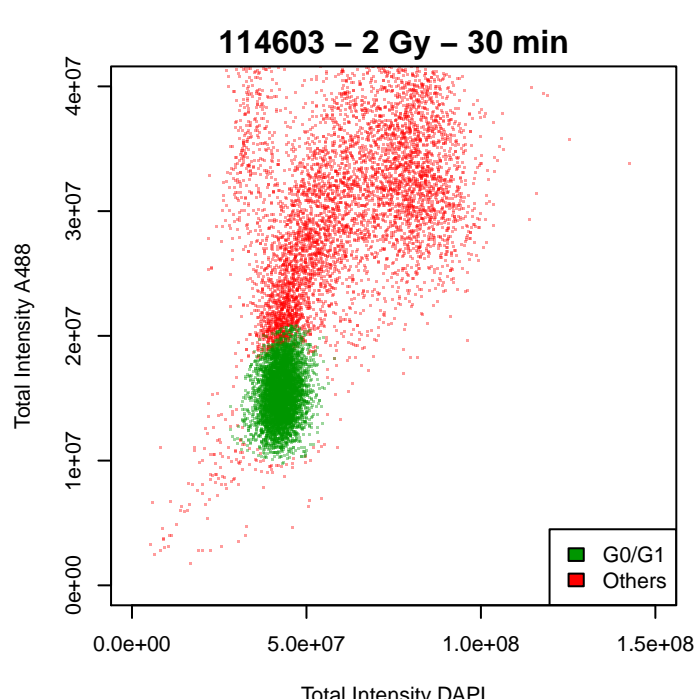

Q

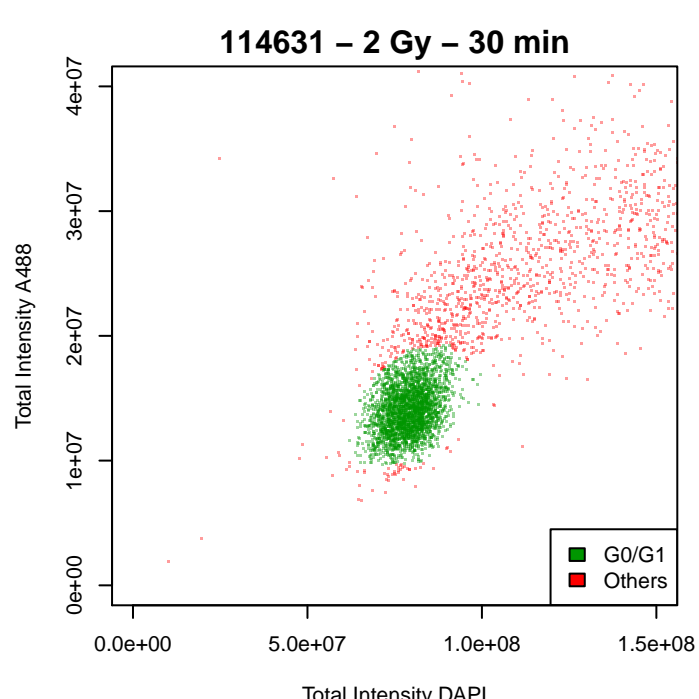

R

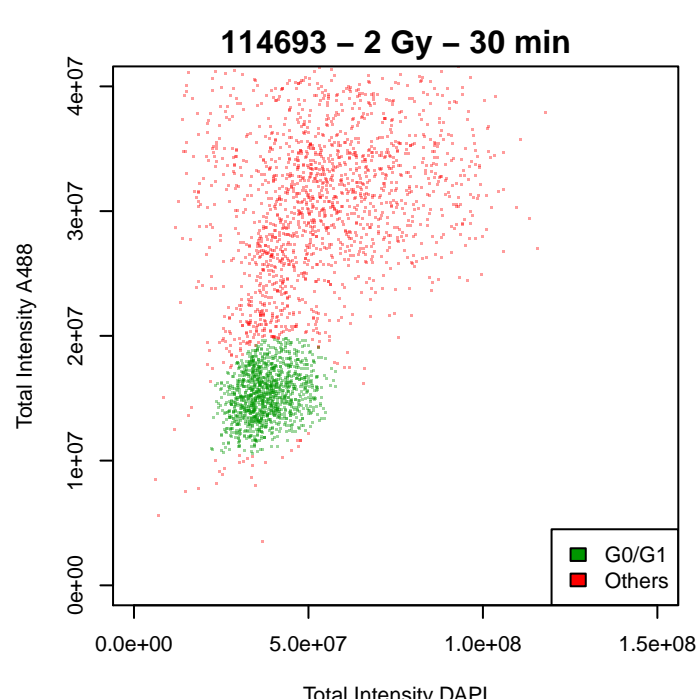

Supplement: S1 Fig — The integrated intensities of DAPI and Alexa Fluor® 488 correspond respectively to the DNA content and level of gamma-H2AX in the whole nucleus of each analysed cell. Green dots indicate a well-defined population of nuclei mainly in the G0/G1 phase of the cell cycle used to performe the RIF analysis. Red dots correspond to nuclei not used for the RIF analysis. (A) to (R): the green subpopulation could be easily discriminated in all conditions tested. (PDF) [file pone.0145786.s001.pdf]

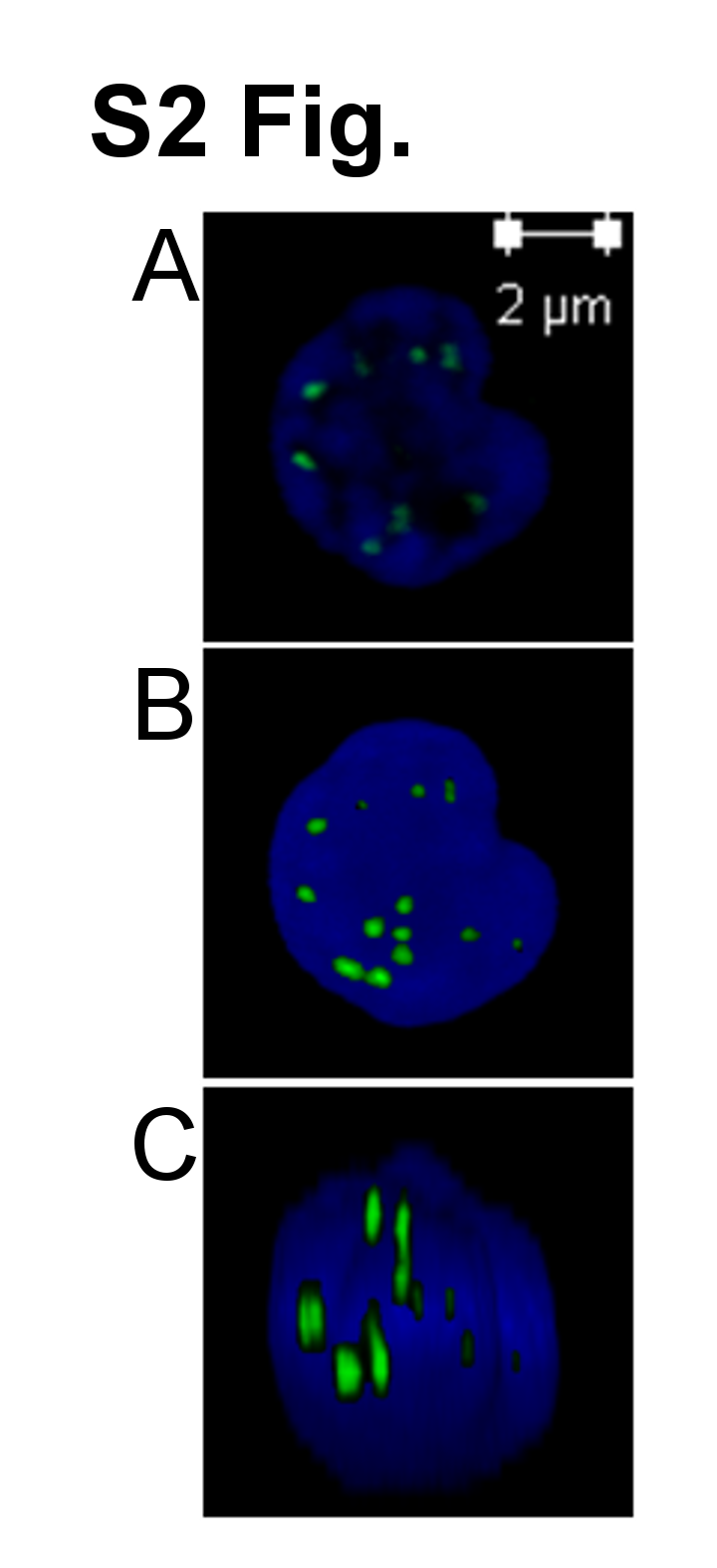

Supplement: S2 Fig — This allows the study of the distribution of foci in the whole volume of irradiated lymphocyte nuclei. (A) Gamma-H2AX foci viewed at the focal plane, as for conventional microscopy. (B) Top view after 3D reconstruction, with confocal microscopy acquisition of 45 layers across the nucleus. The number of foci is higher to that observed at the focal plane. (C) Side view of the 3D reconstruction. In view of the size of the foci, the thickness of the lymphocyte and the depth of field of the x63 objective used, it appears that all foci that can be detected with confocal microscopy cannot be detected at focal plane with conventional microscopy. The picture is representative of all the nuclei examined by confocal microscopy. (TIF) [file pone.0145786.s002.tif]
